# Predicting rice drought-responsive genes via distance-based prototypical graph neural network with path aggregation mechanism

**DOI:** 10.1186/s13007-026-01503-y

**Published:** 2026-03-11

**Authors:** Jing Liu, Hongyan Zhang, Song Wang, Ning Zhang, Xinghui Zhu, Yi Xiao

**Affiliations:** 1 College of Information and Intelligence, Hunan Agricultural University, Changsha, 410128 Hunan China; 2 Department of Interdisciplinary Science and Smart Seed Industry Equipment, Yuelushan Laboratory, Changsha, 410128 Hunan China

**Keywords:** Rice, Drought-responsive gene, Graph neural network, Prototypical network, Path aggregation mechanism

## Abstract

Drought is a primary factor that adversely impacts rice yield and quality. Identifying drought-responsive genes is essential for developing drought-responsive cultivars. Recently, graph neural network methods based on embedding learning have shown considerable success in biological networks. However, challenges still remain in adequately capturing node attribute features, representing topological structures, and addressing class imbalance, which may constrain the model’s predictive capability. To address these issues, we propose a distance-based prototypical graph neural network with path aggregation mechanism (DPGNNPAM) to mine drought-responsive genes in rice. First, we combine gene expression data and protein interaction networks in rice to construct graph-based datasets. Next, we utilize a random walk strategy to generate diverse walk paths and employ a recursive neural network-based path aggregator to encode node attributes along these paths. The prototypical network approach is subsequently employed during training to focus on global information and address the issue of sample imbalance. After that, the weighted similarity is computed by measuring the distance between the node embeddings and the class prototypes. Specifically, class prototypes are representative embeddings that capture the central characteristic of each class. Finally, we transform this value into a predictive probability using the softmax function. Experimental results demonstrate that DPGNNPAM outperforms traditional graph neural network algorithms in identifying drought-responsive genes in rice. Ultimately, we identify 17 candidate genes closely related to drought stress, 12 of which are confirmed in the literature as being involved in the plant’s drought stress response.

**Supplementary Information:**

The online version contains supplementary material available at https://doi.org/10.1186/s13007-026-01503-y.

## Introduction

Drought represents a significant and growing threat to global food security, as it adversely affects plants through mechanisms such as oxidative stress, membrane damage, and photosynthetic inhibition. These harmful effects, coupled with impaired nutrient uptake, ultimately result in stunted growth [16, 39]. Consequently, the identification of drought-responsive genes in rice has become a critical approach for developing new drought-responsive varieties, which are essential for enhancing rice’s environmental adaptability.

With advancements in molecular marker technology and the decreasing cost of genome sequencing, techniques such as genome-wide association studies (GWAS), weighted gene co-expression network analysis (WGCNA), and random walk algorithms, which are rooted in mathematical and statistical analysis, have proven to be highly effective for identifying resistance genes. Yi et al. [44] employed GWAS on recombinant self-crosses of the drought-tolerant Lvhan 1 and drought-susceptible Aixian 1 varieties to identify 10 candidate genes associated with drought response. Although GWAS performs well in identifying common genetic variants, it has limited parsing ability for rare genetic variant detection, which somewhat constrains its effectiveness [27]. Liu et al. [22] used WGCNA to predict 11 candidate genes associated with rice drought stress based on multi-platform gene expression data. However, WGCNA identified gene modules by relying solely on single-transcriptional profiling and did not account for complex interactions across various biological levels. In contrast, Zhu et al. [53] developed a multiplex network that integrated rice protein interaction and gene expression data, employing a restarted random walk algorithm with known drought-responsive genes as seed nodes to identify 13 genes associated with rice drought stress. Building on this, Liu et al. [21] proposed a randomized walking algorithm with multiple restart probabilities, leveraging eigenvector centrality to assess node importance in the network, adjusting restart probabilities accordingly, and ultimately identifying 10 genes that respond to drought stress. This approach synthesizes multi-omics data and shows an improved capacity to uncover complex relationships among genes. Graph neural networks and their variants have been widely used in biology, especially in gene regulatory network analysis and disease-related gene prediction. On this basis, to overcome the limitations of single-omics data in resistance gene prediction, methods integrating multi-omics data have gained prominence as a burgeoning research trend. Wang et al. [41] formulated a novel multi-omics graph convolutional network method that efficiently identifies key biomarkers by integrating histology-specific learning and cross-omics association learning. Yuan et al. [48] created a deep learning model scMGATGRN, utilizing a multi-view graph attention network to infer gene regulatory networks (GRNs) from single-cell RNA sequencing data, which effectively captured the complex feature information in GRNs. Shen et al. [33] developed NPI-GNN, a method combining GraphSAGE and top-k pooling techniques, which integrates multi-view information to efficiently aggregate features from neighboring nodes across different scales for accurate prediction of ncRNA-protein interactions. Although existing graph neural network algorithms have been successful in gene prediction tasks, they still face challenges when applied to imbalanced datasets. These models tend to prioritize features from the majority class, and their limited capacity to identify minority class samples leads to suboptimal performance, especially on minority class data, ultimately hindering the models’ generalization ability.

Based on the above observations, this paper summarizes several limitations of current graph neural networks in gene prediction tasks, such as insufficient consideration of category imbalance, neglect of the long-range dependencies between genes, and a suboptimal capacity for learning the topological structure within the network. To tackle these challenges, we propose a novel algorithm, DPGNNPAM (Distance-Based Prototypical Graph Neural Network with Path Aggregation Mechanism). The main contributions of this paper are summarized as follows:

- Utilizing gene expression profiles in conjunction with protein-protein interaction network data, we constructed a graph-based dataset to facilitate the exploration and identification of genes related to drought-responsive in rice.
- DPGNNPAM introduces a random-walk-based path aggregation mechanism to replace the conventional neighbor aggregation method. This approach effectively captures both node attribute features and topological structures within gene networks, yielding high-quality node embeddings for subsequent prediction tasks.
- DPGNNPAM incorporates a prototype-driven learning mechanism to address category imbalance, thereby enhancing the model’s sensitivity to minority classes and significantly boosting its overall predictive performance.
- Compared to traditional graph neural networks (e.g., GCN, GAT, GraphSAGE), our DPGNNPAM model demonstrates superior information mining capabilities for predicting drought-responsive genes, achieving accuracy and precision scores of 0.8987 and 0.9173, respectively.

## Materials and methods

### Datasets

#### Gene expression profiling data

In this study, we selected 31 rice samples under water-abundant conditions and 52 rice samples subjected to drought conditions. Gene names were standardized according to the Rice Annotation Project Database (RAP-DB), with examples such as ’Os01g200001’. Gene data that could not be mapped to the RAP-DB format were subsequently removed. Consequently, we constructed a gene expression matrix with 28,865 genes and 83 samples. The relevant experimental data can be accessed at Figshare [23, 49].

#### Protein interaction network

In this study, we retrieved a total of 15,535,360 rice protein interaction pairs from the STRING database (version 11.5), which was accessed on 18 October 2024 [32]. To ensure the reliability of the interaction data, only those interaction pairs with a combined score greater than 0.4 were retained, resulting in a subset of 2,369,663 interaction pairs. To maintain consistency in nomenclature between the gene expression profiling data and the protein interaction network, we obtained 33,675 rice gene-protein correspondences from RAP-DB (https://rapdb.dna.affrc.go.jp/, accessed on 20 October 2024) [11].

#### Known drought-responsive genes

In this study, we retrieved 400 breeding-associated drought-responsive genes from the National Rice Data Center (https://www.ricedata.cn/, accessed on June 1, 2024). These genes were defined as positive samples in the graph-based data to validate the graph neural network model. After removing 4 genes that could not be matched during name standardization and excluding 21 genes that were absent from the protein–protein interaction network, 375 drought-responsive genes were finally retained for analysis.

#### Constructing graph datasets

**Fig. 1:**
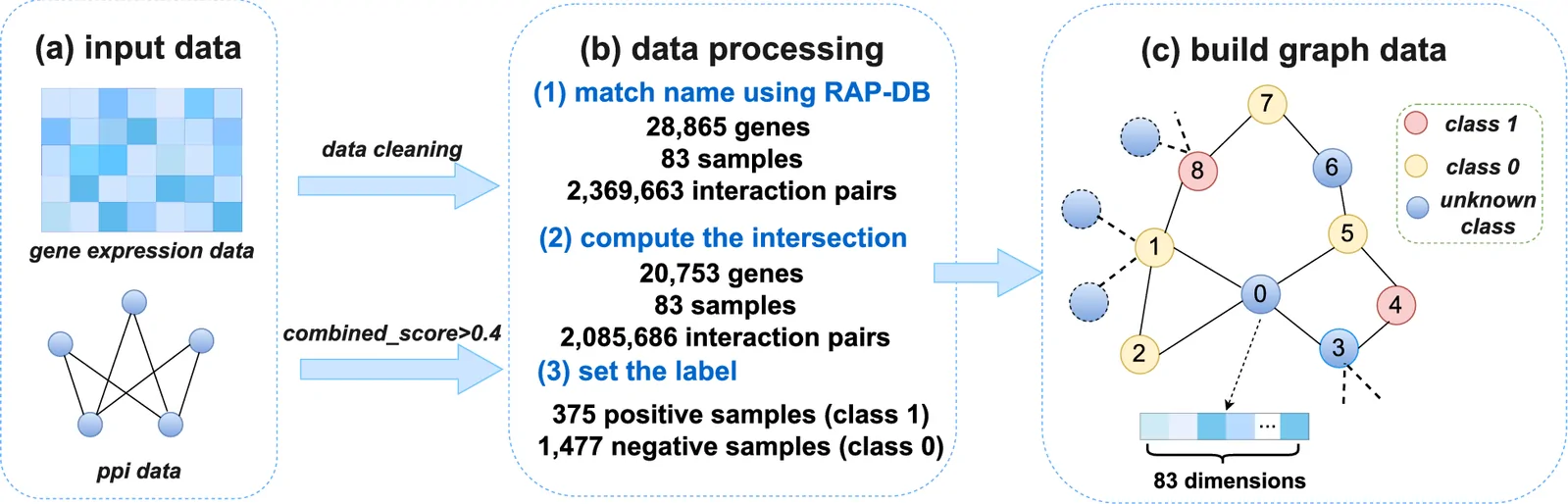
Construct graph-based data. First, we preprocessed the gene expression data and the protein-protein interaction data separately. Then, we used rap-db to match the names of genes and proteins; calculated the intersection of both datasets; and designated specific categories for some genes. Finally, we constructed the graph-based data by taking genes as nodes and the interaction relationships between proteins as connections

We intersected the genes from the gene expression profiles with those in the protein interaction network, resulting in a total of 20,753 nodes and 2,085,686 edges in the graph. Subsequently, we assign the expression levels of each gene across 83 samples as attribute values to their corresponding nodes. Experimentally validated drought-responsive genes were retrieved from the National Rice Data Center, and the corresponding nodes in the graph were designated as positive samples (class 1). The top 3,000 nodes with the lowest variance across 83 samples were selected from the 20,753 nodes, while known drought-responsive genes and their interacting genes in the rice protein-protein interaction network were excluded. The remaining nodes, considered irrelevant to drought stress, were designated as negative samples (class 0). The data construction process is shown in Fig. 1.

### Node embedding learning based on random walk

The mining of genes potentially involved in rice responses to drought stress can be effectively framed as a gene classification problem. We treat gene expression values across 83 samples as node attributes and protein interaction relationships as the connections between nodes. By using network embedding methods, we can integrate node attributes and the topological structure of the network, generating gene embedding vectors that help in the comprehensive extraction of rice features. To generate semantically enriched node embeddings, this study introduces the random-walk sampling technique, extending traditional neighborhoods into path-based neighborhoods. Random walks with two parameters *p* and *q* from well-established Node2Vec are employed to simulate breadth-first search and depth-first searches [12]. The transfer probability of the random walk is set as follows:

1 $$\begin{aligned} \alpha _{\textrm{uv}}(t,x) = \left\{ \begin{array}{ll} \frac{1}{u}, & d_{tx} = 0, \\ 1, & d_{tx} = 1, \\ \frac{1}{v},& d_{tx} = 2. \end{array} \right. \end{aligned}$$

here $d_{tx}$ is a topological indicator based on the connectivity between the previous node *t* and the candidate next node *x*: $d_{tx}=0$ if *x=t* (return), $d_{tx}=1$ if *x* is a neighbor of *t*, and $d_{tx}=2$ if *x* is not directly connected to *t* (distant). The parameters *u* and *v* modulate the random walk’s tendency to remain local or explore further afield. When *u<1*, the random walk is biased toward returning to the previous node (return quickly), which is suitable for capturing homogeneous information similar to the target node; when *v<1*, random wandering tends to wander deeper, suitable for capturing heterogeneous information further away.

The *K*-hop paths generated by the random walk preserve both the node attributes and the sequential order of visited nodes. In this study, a Gated Recurrent Unit (GRU) [43] is employed to encode each path into a fixed-dimensional embedding vector. The path embedding is computed as follows:

2 $$\begin{aligned} h_p^{(l)} = f_{\text {path}}^{(l)}(p) = f_\theta \left( h_{n_1}^{(l)}, h_{n_2}^{(l)}, \ldots , h_{n_k}^{(l)} \right) , \end{aligned}$$

where $h_p^{(l)}$ denotes the embedding of path *p* at the *l*-th layer. For each target node $v_i$, we collect the set of path embeddings $\{ h_p^{s} \mid p \in \mathcal {P}_i^s \}$, where $\mathcal {P}_i^s$ denotes the collection of all sampled paths starting from $v_i$ under strategy *s*. In this study, the sampling strategy *s* encompasses two distinct approaches: depth-first search (DFS) and breadth-first search (BFS), which enable the model to capture both deep and wide structural patterns in the graph.

To aggregate these path embeddings into a comprehensive representation for $v_i$, we employ an attention mechanism that learns the relative importance of each path. The attention weight for a path $p \in \mathcal {P}_i^s$ is defined as:

3 $$\begin{aligned} e_p = \text {LeakyReLU}\left( w_p^T \cdot h_p^s \right) , \end{aligned}$$

where $w_p$ is a learnable attention parameter and $e_p$ is the unnormalized importance score. The normalized attention weight $\alpha _p$ is computed via softmax:

4 $$\begin{aligned} \alpha _p = \frac{\exp (e_p)}{\sum _{r \in \mathcal {P}_i^s} \exp (e_r)}. \end{aligned}$$

After that, the node embedding $h_i^s$ under strategy *s* is computed as a weighted sum of all path embeddings originating from node $v_i$:

5 $$\begin{aligned} h_i^s = \sum _{p \in \mathcal {P}_i^s} \alpha _p \cdot h_p^s. \end{aligned}$$

Eventually, the final node embeddings were obtained from all S strategies. Specifically, the final node embedding $h_i$ is generated by concatenating the embeddings from each individual strategy:

6 $$\begin{aligned} h_i = \bigoplus _{s \in S} h_i^s, \end{aligned}$$

where *S* denotes the set of sampling strategies, and *⊕ * represents the concatenation operation. This fusion enables the model to leverage complementary information captured by different strategies, leading to richer and more robust node representations.

### Prototypical network for loss balancing

During the data processing phase, we constructed graph-based data and identified a significant imbalance between positive and negative samples, with negative samples being approximately four times more than positive ones. This imbalance may adversely affect the performance of the model. In deep learning methods, the gradient is often dominated by majority class samples, making the model more effective at learning from the majority class while neglecting the minority class [54]. To deal with this issue, we use a prototypical network to balance the training loss between the majority and the minority classes [38]. Of the labeled nodes, 80% were allocated for training and 20% for testing through stratified random sampling, which preserved the overall class distribution in both sets. The training set is further divided into a query set $Q = \{ Q_c \mid c \in C \}$ and a support set $S = \{ S_c \mid c \in C \}$. The query set is used for evaluating classification, while the support set is used to learn feature representations and construct class prototypes. In prototypical network, a class prototype represents the global characteristics of the nodes belonging to the same class [18]. In our setup, the number of class prototypes *C* is set to 2, corresponding to the two categories of genes: drought-tolerant and non-drought-tolerant. The prototype $P_c$ for class *c* is computed as:

7 $$\begin{aligned} P_c = PROTO(H^{S_c}) = PROTO(\{ h_i \mid v_i \in S_c \}), \end{aligned}$$

where *PROTO(· )* denotes the aggregation operation used to compute the prototype from the support set. In this study, mean pooling is adopted:

8 $$\begin{aligned} P_c = \frac{1}{|S_c|} \sum _{v_i \in S_c} h_i. \end{aligned}$$

Thus, each class prototype $P_c$ serves as the representative embedding for class *c* in the metric space.

### Predictive probability computation

Once class prototypes are obtained, we map each node into a distance metric space to capture its relative position with respect to all class prototypes. For a node embedding $h_i \in \mathbb {R}^d$, we first compute its difference to each class prototype:

9 $$\begin{aligned} g_i^c = h_i - P_c, \quad c \in \{1, \dots , C\}. \end{aligned}$$

We then concatenate all the difference vectors to form a comprehensive distance-aware representation:

10 $$\begin{aligned} G_i = \left[ g_i^1 \Vert g_i^2 \Vert \cdots \Vert g_i^C \right] \in \mathbb {R}^{C \cdot d}, \end{aligned}$$

where * ‖ * denotes vector concatenation. A learnable linear transformation $f_2: \mathbb {R}^{C \cdot d} \rightarrow \mathbb {R}^{d'}$ is then applied to extract meaningful distance information: $\tilde{G}_i = f_2(G_i)$. Let $Q = \{1, \dots , N_q\}$ and $S = \{1, \dots , N_s\}$ denote the sets of query and support nodes, respectively. We collect their transformed representations into matrices:

11 $$\begin{aligned} \tilde{G}^Q = \begin{bmatrix} \tilde{G}_1^Q \\ \tilde{G}_2^Q \\ \vdots \\ \tilde{G}_{N_q}^Q \end{bmatrix} \in \mathbb {R}^{N_q \times d'}, \quad \tilde{G}^S = \begin{bmatrix} \tilde{G}_1^S \\ \tilde{G}_2^S \\ \vdots \\ \tilde{G}_{N_s}^S \end{bmatrix} \in \mathbb {R}^{N_s \times d'}, \end{aligned}$$

where each row corresponds to one node’s transformed distance-aware embedding.

The similarity between query and support nodes is computed via matrix multiplication followed by softmax:

12 $$\begin{aligned} F = \textrm{softmax}\left( \tilde{G}^Q (\tilde{G}^S)^\top \right) \in \mathbb {R}^{N_q \times N_s}, \end{aligned}$$

where $F_{ij}$ represents the similarity score (interpreted as probability) between the *i*-th query node and the *j*-th support node.

Since multiple support nodes may belong to the same class *c*, we aggregate their similarities to obtain class-level probabilities. Then the probability that query node *i* belongs to class *c* can be expressed as:

13 $$\begin{aligned} F_{i,c} = \frac{1}{|S_c|} \sum _{j \in S_c} F_{ij}. \end{aligned}$$

Finally, the predicted probability distribution over classes for the *i*-th query node is $F_i = [F_{i,1}, \dots , F_{i,C}]$, and the classification loss is defined as follow:

14 $$\begin{aligned} \ell _{\textrm{class}} = \frac{1}{C}\sum _{c=1}^{C} \ell (F_{i,c}, c), \end{aligned}$$

**Fig. 2:**
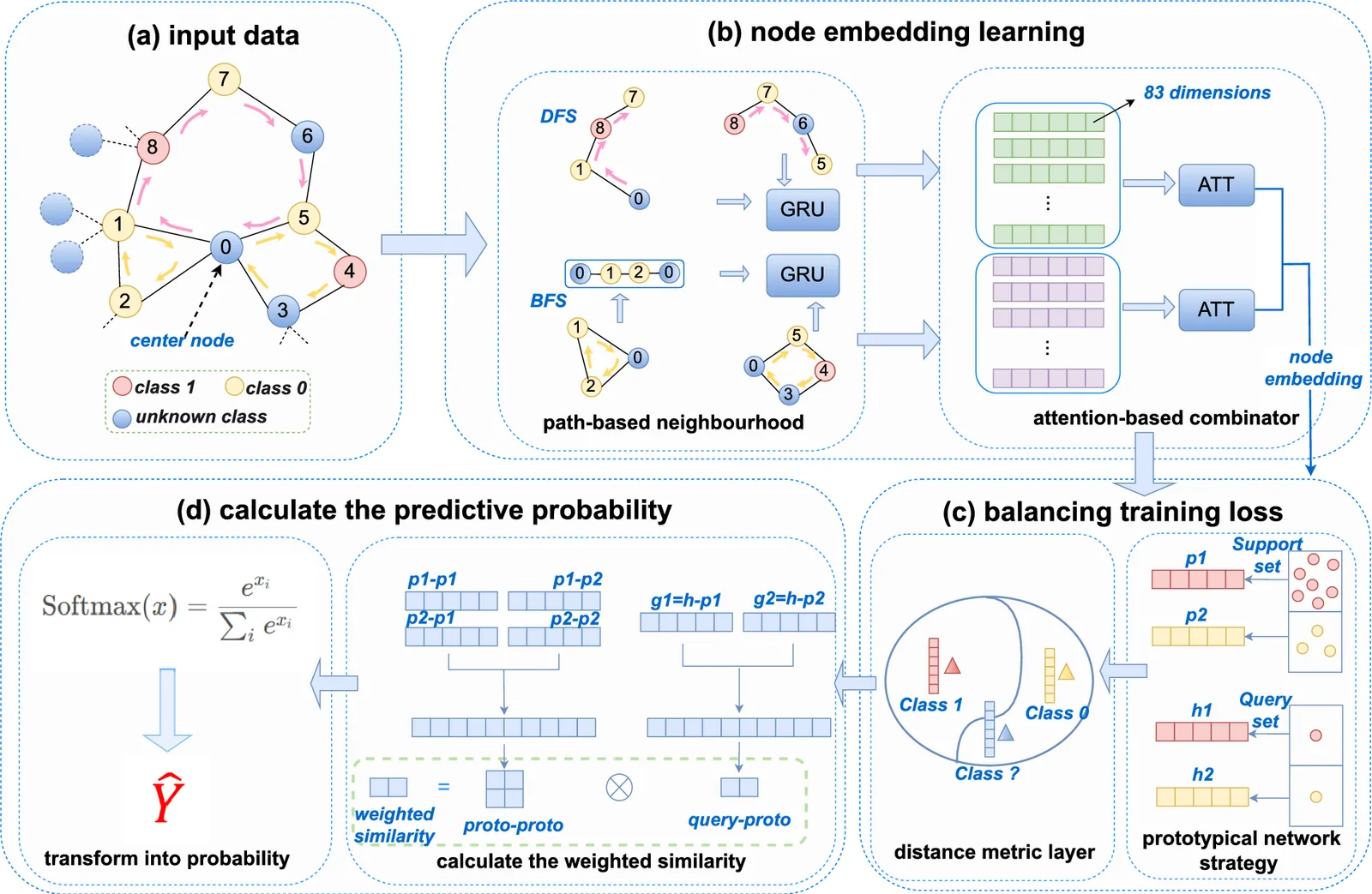
Flowchart of the algorithm. The algorithm takes gene expression profile data and protein interaction network as inputs. It generates the probability of each gene being a drought-responsive gene as outputs. Initially, we use a graph neural network with path aggregation mechanism to assign different weights to various paths. Subsequently, the embedding vectors are mapped to the distance metric space using the prototypical network strategy. To evaluate the distance between the query nodes and the class prototypes, we compute the weighted similarity. Ultimately, the weighted similarity is converted into predicted probability values through the application of the softmax function

$\ell (\cdot ,\cdot )$ is a loss function to measure the difference between predictions and ground-truth labels. The overall algorithm process is shown in Fig. 2.

### Design of comprehensive loss functions

Based on intuitive understanding, class prototypes of different categories should exhibit significant differences, while node representations within the same category should be as similar as possible. To ensure that prototypes of different classes are represented distinctly, the following objective function is introduced to minimize the similarity of representations between prototypes of different classes:

15 $$\begin{aligned} \ell _{ssl_p}=\sum _{i=1}^{C}{\sum _{j=1}^{C}{(SIM_{ij})-tr(SIM)}}, \end{aligned}$$

16 $$\begin{aligned} \textrm{SIM}_{ij} = \frac{p_i^T p_j}{\Vert p_i\Vert \cdot \Vert p_j\Vert }, \end{aligned}$$

where $p_i$ and $p_j$ are the prototype vectors of class *i* and class *j*. $\Vert p_i\Vert$ and $\Vert p_j\Vert$ represent their respective magnitudes. $\textrm{SIM}_{ij}$ denotes the cosine similarity between prototype $p_i$ and $p_j$, and $\textrm{SIM}$ is the *C × C* similarity matrix constructed from all pairwise similarities of class prototypes. Here, *tr*(*SIM*) denotes the trace of the similarity matrix, which is the sum of its diagonal elements. By minimizing the loss function $\ell _{ssl_p}$, the model reduces the similarity between prototypes of different categories, encouraging the prototype vectors of each category to be as far apart from each other as possible in the representation space.

To enhance intra-class similarity, we introduce a smoothing loss that encourages neighboring nodes of the same class to have similar distances to their class prototype:

17 $$\begin{aligned} \ell _{ssl_s} = \sum _{v_i \in \mathcal {V}} \sum _{v_j \in \mathcal {N}_i} \left( \frac{g_i}{\sqrt{d_i}} - \frac{g_j}{\sqrt{d_j}} \right) ^2, \end{aligned}$$

where $g_i = \Vert \textbf{h}_i - \textbf{P}_{c_i} \Vert$ is the distance from node $v_i$ to its class prototype, and $d_i$ is the degree of node $v_i$. Here, $\mathcal {N}_i$ denotes the set of neighboring nodes directly connected to $v_i$. This formulation encourages compact and smooth representations within each class. The target function $\ell _{\textrm{ssl}_s}$ becomes small when adjacent nodes exhibit similar distance representations to their class prototypes, thereby promoting both structural smoothness and semantic consistency.

Thus, the model’s global loss function is formulated as the sum of the supervised classification loss and the two self-supervised component losses. The supervised classification loss guides the model to accurately classify labeled data, while the self-supervised component losses enhance its ability to learn and capture the underlying network topology. We train our model by minimizing the total objective loss. The overall objective is expressed as:

18 $$\begin{aligned} \ell = \ell _{\text {class}} + \lambda _1 \ell _{\text {ssl}_p} + \lambda _2 \ell _{\text {ssl}_s}, \end{aligned}$$

where $\ell _{\text {class}}$ is the classification loss previously defined in Eq. (14), $\ell _{\text {ssl}_p}$ and $\ell _{\text {ssl}_s}$ are the self-supervised prototype and smoothness losses, respectively, and $\lambda _1$, $\lambda _2$ are the balancing coefficients. This unified objective enables the model to benefit from both label supervision and structural self-supervision.

### Association analysis

Based on the principle that functionally correlated genes often share similar biological characteristics [52], we utilized protein-protein interaction (PPI) network analysis to identify novel genes potentially involved in drought resistance. To identify high-confidence candidate genes, we employed the following stringent criteria: a gene $G_i$ was selected as a putative drought-responsive gene if it interacted with at least two established drought-responsive genes, and each of those interactions had a *combined_score* *≥ * 0.9. The *combined_score* in STRING integrates multiple evidence channels, including co-expression, experimentally determined interactions, and text mining, providing a robust measure of interaction confidence, and based on this workflow, we identified 17 high-confidence candidate genes.

### Complexity analysis

DPGNNPAM extends DPGNN by incorporating a random-walk-based path aggregation module to enhance node embeddings, while retaining distance metric learning and self-supervised learning from DPGNN. The path aggregation module includes three components: an RNN-based path aggregator, an attention-based intra-strategy combinator, and an inter-strategy combinator. For each node, *r* random walks of length *L* are sampled. The RNN-based path aggregator (GRU) processes the sequence of *L* nodes, each with feature dimension *d*, and outputs embeddings of hidden dimension $d_h$. The time complexity of this step is $O(r \cdot L \cdot |V| \cdot d \cdot d_h )$. Next, the intra-strategy combinator aggregates the embeddings of multiple paths per node within each sampling strategy using attention. Assuming attention computation scales linearly with the number of paths *r* and hidden dimension $d_h$, the complexity is $O(r \cdot |V| \cdot d_h )$. Finally, the inter-strategy combinator concatenates the embeddings from all strategies (|*S*| strategies) for each node. Since concatenation is a memory operation, it introduces negligible additional computational cost, effectively $O(|V| \cdot |S| \cdot d_h)$.

Combining these, the total additional time complexity of the path aggregation module is $O(r \cdot L \cdot |V| \cdot d \cdot d_h + r \cdot |V| \cdot d_h)$, which scales linearly with the number of nodes for practical *r* and *L*. Other components, including distance metric learning and self-supervised learning, follow the same complexity analysis as in DPGNN [38]. Regarding parameters, the path aggregation module introduces additional weights from the GRU and attention layers, scaling with $O(d \cdot d_h + d_h^2)$ per path aggregator. Distance metric learning adds $O(d_{C}' \cdot d'')$ parameters, where $d_{C}'$ is the concatenated dimension of class prototype embeddings and *d''* is the dimension after linear transformation. These additional parameters are typically smaller than the GNN encoder parameters *O(d' · d'')*. Overall, DPGNNPAM remains computationally efficient while providing richer node representations.

## Experimental setup and results

### Baseline methods

In this study, we compared our proposed approach with the following baseline tasks: (1) the traditional graph neural network model GCN [17], (2) GraphSAGE, which operates under the homogeneity assumption [13], (3) Graph Attention Network [37], (4) Path Aggregation-based graph neural network, RAW-GNN [14], (5) Distance-based prototype graph neural network DPGNN [38], and (6) the bias-variance decomposition-based model ReVar [46].

All models were implemented within a unified framework to ensure fair and reproducible comparisons. A two-layer GNN architecture was adopted with a hidden dimension of 32 and a dropout rate of 0.5. GraphSAGE employed the mean aggregator, and GAT used 2 attention heads. The Adam optimizer was used with a learning rate of 0.01, along with a parameter group-wise optimization strategy: weight decay of $5 \times 10^{-4}$ was applied to the first GNN layer, and no weight decay to the second. Training ran for a maximum of 200 epochs, with early stopping enabled after the first 100 epochs. Node features were normalized before input. For RAW-GNN and DPGNN, long random walks were set to 10 steps and short walks truncated to 4 steps, with 4 independent long walks generated per node to enrich structural context. In models with multi-component loss functions, both inter-class and intra-class loss terms were assigned a weight of 10. For ReVar, we followed the hyperparameter settings as described in the original study, including the bias-variance decomposition objective and associated loss balancing coefficients. This standardized setup ensures performance differences stem primarily from model architectures.

### Comparison of model performance

To fully evaluate the performance of the DPGNNPAM algorithm in predicting drought-responsive genes in rice. We conducted 5 independent runs, each consisting of 200 training epochs. The results were averaged to ensure the stability and reliability of the experimental outcomes. Finally, we used several evaluation metrics, including Accuracy, weighted F1, Precision, ROC AUC (AUC), and PR AUC (AP), to provide a comprehensive assessment of the model’s effectiveness in predicting drought-responsive genes. The threshold was set to 0.6 to minimize false positives and prioritize high-confidence predictions. The evaluation metrics are as follows:

19 $$\begin{aligned} \text {Accuracy} = \frac{\text {TP} + \text {TN}}{\text {TP} + \text {TN} + \text {FP} + \text {FN}}, \end{aligned}$$

20 $$\begin{aligned} \text {Precision} = \frac{\text {TP}}{\text {TP} + \text {FP}}, \end{aligned}$$

21 $$\begin{aligned} \text {F1-score} = 2 \times \frac{\text {Precision} \times \text {Recall}}{\text {Precision} + \text {Recall}}, \end{aligned}$$

22 $$\begin{aligned} \text {weighted F1} = \frac{1}{N} \sum _{i=1}^{C} n_i \cdot \text {F1}_i, \end{aligned}$$

23 $$\begin{aligned} \text {ROC-AUC} = \int _{0}^{1} \text {ROC Curve}, \end{aligned}$$

24 $$\begin{aligned} \text {PR-AUC} = \sum _{i=1}^{n} P(i) \cdot \Delta R(i), \end{aligned}$$

where TP (TN) denotes the number of true positive (negative) instances, and FP (FN) denotes the number of false positive (negative) instances, respectively. This weighted F1 averages the per-class F1-scores, weighted by the number of true instances in each class. ROC-AUC represents the area under the Receiver Operating Characteristic (ROC) curve. PR-AUC represents the area under the Precision-Recall (PR) curve. In the PR-AUC formula, *P*(*i*) denotes the precision at the * i *th threshold, and * Δ R(i) * represents the change in recall between the * i *th and * (i-1) *th threshold. The final experimental results are presented in Table 1.

**Table 1:** Comparison of the performance of the models

	Weighted F1	Accuracy	Precision	ROC-AUC	PR-AUC
GCN	0.8098±0.0834	0.8054±0.1044	0.6447±0.2468	0.8593±0.0328	0.6794±0.0998
GraphSage	0.8173±0.1090	0.8124±0.1269	0.7124±0.3338	0.8273±0.1058	0.6230±0.2445
GAT	0.8219±0.0393	0.8302±0.0338	0.6134±0.1334	0.8551±0.0277	0.5917±0.0698
DPGNN	0.8610±0.0127	0.8582±0.0155	0.6384±0.0461	0.8721±0.0155	0.7240±0.0483
RAW-GNN	0.7374±0.0325	0.8595±0.0262	0.6010±0.0602	0.8483±0.0067	0.7788±0.0206
ReVar(GCN)	0.8770±0.0077	0.8906±0.0059	0.9083±0.0158	0.7375±0.0206	0.6962±0.0077
ReVar(GAT)	0.8741±0.0037	0.8889±0.0030	0.9138±0.0101	0.7848±0.0218	0.7113±0.0140
ReVar(Sage)	0.8420±0.0200	0.8658±0.0138	0.8682±0.0297	0.7027±0.0574	0.6038±0.0672
DPGNNPAM	0.8862±0.0262	0.8987±0.0186	0.9173±0.0538	0.8759±0.0292	0.7934±0.0235

Our proposed DPGNNPAM algorithm achieves state-of-the-art performance across all evaluation metrics, consistently outperforming both classical GNNs and the imbalance-aware ReVar framework.

Notably, ReVar(GCN) and ReVar(GAT) achieve strong performance, ranking second and third in weighted F1 and accuracy, with ReVar(GAT) achieving the highest precision (0.9138±0.0101) among all baselines. This highlights the effectiveness of bias-variance decomposition in mitigating class imbalance in node classification. However, DPGNNPAM still surpasses both variants, improving weighted F1 by 1.05% and 1.38%, respectively, with comparable standard deviations, indicating similar levels of stability.

In contrast, traditional GNNs exhibit suboptimal performance due to their reliance on neighborhood aggregation mechanisms that tend to favor majority-class nodes under extreme label imbalance. DPGNN improves upon these by introducing prototype-based learning, achieving better weighted F1 and ROC-AUC, yet still lags behind DPGNNPAM in precision and PR-AUC. RAW-GNN, which incorporates path-based structural information, achieves the second-highest PR-AUC (0.7788±0.0206), indicating its strength in handling imbalanced precision-recall trade-offs, but is outperformed by DPGNNPAM in all other metrics.

The performance gain of DPGNNPAM can be attributed to its use of random-walk-based neighborhood sampling, which enhances structural diversity, combined with a prototype-based learning mechanism that strengthens representation learning for minority-class nodes. This design helps mitigate the impact of label imbalance and improves prediction reliability.

### Ablation study

We conduct comprehensive ablation studies to evaluate the contributions of key components in DPGNNPAM. The variants are enumerated consistently as follows:

1. **Excluding DFS (BFS-only):** Restricting the random walk strategy to pure Breadth-First Search to evaluate the sufficiency of local structural information.
2. **Excluding BFS (DFS-only):** Restricting the random walk strategy to pure Depth-First Search to evaluate the sufficiency of local structural information.
3. **Excluding Random Walk (GCN):** Replacing random walks with Graph Convolutional Network (GCN)-based neighborhood aggregation to verify the necessity of global information capturing through walks.
4. **Excluding** $\ell _{ssl_s}$: Incorporating compound supervision signals by evaluating $\ell _{class} + \ell _{ssl_p}$ to assess the effect of structure-level self-supervision.
5. **Excluding** $\ell _{ssl_p}$: Incorporating compound supervision signals by evaluating $l_{class}+l_{ssl_s}$ to assess their effects on the model’s discriminative power.
6. **Excluding** $\ell _{ssl_s}+\ell _{ssl_p}$: Using only the classification loss $\ell _{class}$ as the baseline reference.
7. **Excluding Prototypical Network (MLP):** Substituting the prototypical network with a standard Multi-Layer Perceptron (MLP) classifier to analyze the superiority of the metric-learning framework.

**Table 2:** Results of ablation experiments

No	DFS	BFS	Random	$\ell _{\textrm{ssl}_s}$	$\ell _{\textrm{ssl}_p}$	$\ell _{\textrm{ssl}_s}$ $+\ell _{\textrm{ssl}_p}$	Prototypical	Weighted F1	Accuracy	Precision	ROC-AUC	PR-AUC
			walk				network					
1	*× *	*\checkmark *	*\checkmark *	*\checkmark *	*\checkmark *	*\checkmark *	*\checkmark *	0.8596±0.0139	0.8782±0.0092	0.9168±0.0643	0.8062±0.0564	0.7463±0.0406
2	*\checkmark *	*× *	*\checkmark *	*\checkmark *	*\checkmark *	*\checkmark *	*\checkmark *	0.8662±0.0275	0.8771±0.0279	0.8329±0.1610	0.8408±0.0397	0.7197±0.0832
3	*\checkmark *	*\checkmark *	*× *	*\checkmark *	*\checkmark *	*\checkmark *	*\checkmark *	0.8098±0.0834	0.8054±0.1044	0.6447±0.2468	0.8593±0.0328	0.6794±0.0998
4	*\checkmark *	*\checkmark *	*\checkmark *	*× *	*\checkmark *	*\checkmark *	*\checkmark *	0.8409±0.1319	0.8415±0.1512	0.8320±0.2914	0.8389±0.0635	0.7442±0.1618
5	*\checkmark *	*\checkmark *	*\checkmark *	*\checkmark *	*× *	*\checkmark *	*\checkmark *	0.8472±0.0195	0.8523±0.0414	0.6971±0.1544	0.8070±0.0184	0.6672±0.0446
6	*\checkmark *	*\checkmark *	*\checkmark *	*\checkmark *	*\checkmark *	*× *	*\checkmark *	0.8822±0.0966	0.8113±0.1186	0.7044±0.2359	0.7829±0.0574	0.6154±0.0580
7	*\checkmark *	*\checkmark *	*\checkmark *	*\checkmark *	*\checkmark *	*\checkmark *	*× *	0.8070±0.0966	0.8938±0.0109	0.8781±0.0177	0.8457±0.0170	0.7508±0.0242
8	*\checkmark *	*\checkmark *	*\checkmark *	*\checkmark *	*\checkmark *	*\checkmark *	*\checkmark *	0.8862±0.0262	0.8987±0.0186	0.9173±0.0538	0.8759±0.0292	0.7934±0.0235

In this section, we carefully evaluate the contribution of each component through well-designed experiments. The comprehensive results are visualized in Table 2, demonstrating the performance variations across different metrics for each ablated variant. As seen in the table, the DPGNNPAM model outperforms all other variants in every metric, highlighting the importance of its integrated components. Specifically, removing the random walk causes a significant drop in performance, particularly in weighted F1, which decreases from 0.8862 to 0.8098, emphasizing the critical role of this mechanism in capturing long-range dependencies. The absence of DFS and BFS leads to a slight decrease in performance, suggesting that these search strategies contribute to the model’s robustness. The removal of the prototypical network results in a more pronounced decline, with the weighted F1 dropping to 0.8070, underscoring the necessity of this component in accurately modeling class-specific features. Interestingly, ablating either $\ell _{ssl_s}$ or $\ell _{ssl_p}$ alone yields worse weighted F1 performance than ablating both–suggesting that using only one introduces an imbalanced inductive bias in representation learning (e.g., enforcing intra-class compactness without inter-class separation, or vice versa), which degrades performance more than the baseline model with no self-supervised regularization. This counterintuitive result highlights that the effectiveness of these self-supervised components stems not from their individual contributions, but from their complementary synergy: only when jointly applied do they shape a well-structured embedding space that improves robustness under class imbalance. Consequently, the full model–equipped with both $\ell _{ssl_s}$ and $\ell _{ssl_p}$ –achieves the best overall performance. In conclusion, the DPGNNPAM model, by combining multiple advanced strategies such as random walk, prototypical network, and self-supervised components, outperforms the ablated variants by capturing complex relationships and improving the prediction of drought-responsive genes in rice.

### Predicting drought-responsive genes in rice

We applied the DPGNNPAM model to predict unknown genes, ranking all genes predicted as positive category in descending order based on their probability values. The top 2,000 genes were selected for association analysis to identify potential genes associated with drought stress.

### Gene ontology enrichment analysis

**Fig. 3:**
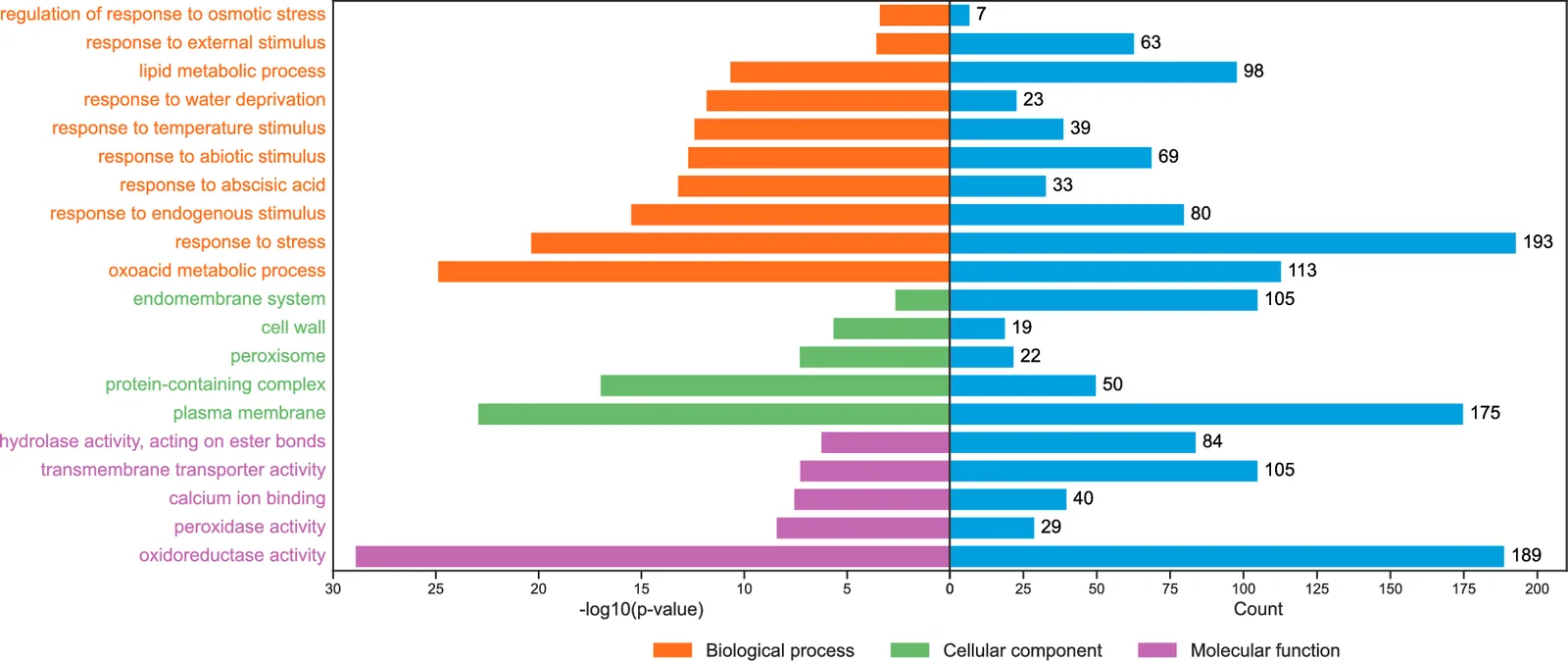
Gene ontology enrichment analysis of the top 2,000 genes with the highest predicted probabilities. The results are categorized by Cellular Component, Molecular Function, and Biological Process

In this study, gene ontology enrichment analysis was conducted on the top 2,000 nodes with the highest predicted probabilities using the Gene Ontology Resource (https://www.geneontology.org/). Gene Ontology Resource, which was embedded with the PANTHER knowledge, providing comprehensive information on the evolution of protein-coding gene families [34]. The selected genes were categorized into three functional categories, including cellular component, molecular function, and biological process, according to the Gene Ontology(GO) taxonomy [8]. A smaller p-value indicated the higher enrichment of a gene in a specific GO category. As illustrated in the Fig. 3, we have selected some of the entries of interest with p-values below 0.01 for presentation.

The results of the enrichment analysis revealed several significant biological findings. In the biological process category, the predicted potential genes were notably enriched in processes related to abiotic stress, particularly in the regulation of osmotic stress response, response to water deficit conditions, organic acid metabolic processes, and response to oxygenated compounds. These findings suggested that these genes may contribute to enhancing plant resilience to environmental stresses, such as water and oxidative stress. In the molecular function category, significantly enriched entries included oxidoreductase activity, calcium binding, and peroxidase activity, which were crucial for the physiological response of plants when encountering adversity, especially in the maintenance of redox homeostasis and cellular signaling processes. Meanwhile, in the category of cellular components, the enriched entries were mainly focused on structures such as cell wall synthesis, transferase complexes, and intracellular membrane-bound organelles, indicating that these genes may be involved in key functions such as structural repair and material transport in plant cells. The results of these enrichment analyses further validated the effectiveness of the prediction method employed in this study.

### Identifying drought-responsive genes via association analysis

**Fig. 4:**
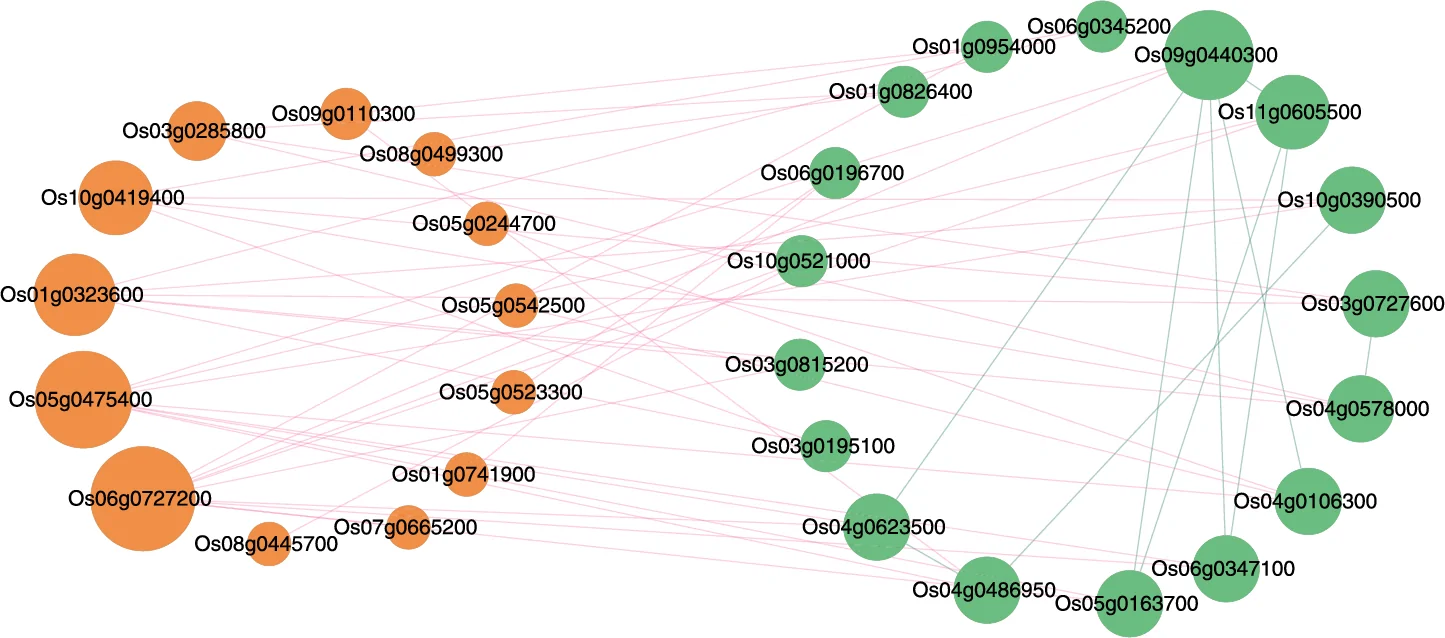
Results of association analysis. Thereinto, orange nodes on the left side indicate known drought-responsive genes, green nodes on the right side indicate predicted potential drought-responsive genes, light pink lines indicate the association relationship between potential drought-responsive genes and known drought-responsive genes, and light green lines indicate an association between potential drought-responsive genes

In this section, we performed association analysis on the top 2,000 predicted genes. Genes that were associated with at least two known drought-responsive genes and had interaction scores greater than 0.7 were selected as candidate genes. Finally, Cytoscape software was used to visualize the relationships between known drought-responsive genes and potential candidates, as shown in Fig. 4.

After comprehensive association analysis and rigorous screening, we identified 17 potential drought-responsive genes that are closely linked to drought stress. Table 3 provides detailed information about these candidate genes which were retrieved from the STRING database.

**Table 3:** Annotation of candidate genes for drought stress

Candidate genes	Name in UniProt	Annotation
Os06g0196700	A3B9A0	Auxin response factor 16; Auxin response factors (ARFs) are transcriptional factors that bind specifically to the DNA sequence 5’-TGTCTC-3’ found in the auxin-responsive promoter elements (AuxREs)
Os04g0106300	Q7X7N2	Arginase 1, mitochondrial; Catalyzes the hydrolysis of L-arginine to urea and L-ornithine. The latter can be utilized in the urea cycle or as a precursor for the synthesis of both polyamines and proline (By similarity)
Os09g0440300	Q69P84	cDNA clone:001-032-H04, full insert sequence; Belongs to the aldehyde dehydrogenase family
Os10g0521000	Q9FWC1	Probable trehalase; Involved in the regulation of trehalose content by hydrolyzing trehalose to glucose; Belongs to the glycosyl hydrolase 37 family
Os03g0195100	Q6VMN7	Aminotransferase ALD1 homolog; Probable aminotransferase that may generate amino-acid-derived defense signals and be involved in resistance to pathogens. Belongs to the class-I pyridoxal-phosphate-dependent aminotransferase family. LL-diaminopimelate aminotransferase subfamily
Os06g0345200	A0A0P0WW75	Os06g0345200 protein
Os04g0486950	Q7XUG1	Malate synthase; Belongs to the malate synthase family
Os03g0727600	Q10DK7	Aminocyclopropane-1-carboxylate synthase 1; Catalyzes the formation of 1-aminocyclopropane-1-carboxylate, a direct precursor of ethylene in higher plants
Os01g0954000	Q941Y8	Probable NADPH:quinone oxidoreductase 2; The enzyme apparently serves as a quinone reductase in connection with conjugation reactions of hydroquinones involved in detoxification pathways
Os03g0815200	Q75HE6	Probable methylenetetrahydrofolate reductase; The probable reversibility of the MTHFR reaction in plants suggests that they can metabolize the methyl group of 5,10-methylenetetrahydrofolate to serine, sugars, and starch
Os10g0390500	A0A0P0XUE4	Os10g0390500 protein
Os01g0826400	Q6IEQ7	WRKY transcription factor WRKY24; Transcription activator. Interacts specifically with the W box (5’-(T)TGAC[CT]-3’), a frequently occurring elicitor-responsive cis-acting element. Negative regulator of both gibberellic acid (GA) and abscisic acid (ABA) signaling in aleurone cells, probably by interfering with GAM1, via the specific repression of GA- and ABA-induced promoters
Os06g0347100	Q0DCC7	Os06g0347100 protein
Os11g0605500	Q2R1G8	Acyl-coenzyme A oxidase; Belongs to the acyl-CoA oxidase family
Os04g0578000	Q7XQ85	cDNA clone:002-105-C04, full insert sequence
Os04g0623500	Q7FAS1	Peroxisomal (S)-2-hydroxy-acid oxidase GLO3; Photorespiratory enzyme that can exert a strong regulation over photosynthesis, possibly through a feedback inhibition on Rubisco activase. Not required for oxalate accumulation (By similarity)
Os05g0163700	Q75IR2	cDNA clone:J023099O18, full insert sequence

Rice has gradually established a well-developed set of drought stress defense mechanisms during growth, including perception of environmental drought signals, transduction and resolution of the signals in the cell, and transcriptional expression of drought-induced stress-responsive genes [10]. During the drought stress response in rice, key enzymes and transcription factors such as Auxin response factor [9], alginase [5], WRKY transcription factor [45], acyl coenzyme A oxidase [29], and peroxisomal (S)-2-hydroxyacid oxidase GLO3 [19] play crucial roles, which further validates the reliability of our predicted candidate genes.

## Discussion

Literature analysis of the candidate genes listed in Table 3 revealed that 12 of these genes (Os06g0196700, Os04g0106300, Os09g0440300, Os10g0521000, Os03g0195100, Os04g0486950, Os03g0727600, Os01g0954000, Os03g0815200, Os01g0826400, Os11g0605500, and Os04g0623500) were involved in the drought stress response process in plants. Next, we analyzed the mechanism of action of key genes involved in the drought stress response in detail. Auxin Response Factor (Os06g0196700) has been widely reported for its significant role in regulating plant response to drought and salt stress. It does so by modulating soluble sugar content, promoting root development, and maintaining chlorophyll levels, ultimately enhancing plant adaptation to drought conditions [9, 51]. Arginase (Os04g0106300) facilitates nitrogen reactivation by catabolizing arginine into ornithine and urea, and ornithine serves as a precursor for polyamine and proline synthesis [26]. Proline acts as a compatible osmolyte, accumulating at high concentrations without harming cellular macromolecules. This helps prevent membrane damage and protein denaturation under severe drought stress conditions [31]. Aldehyde dehydrogenase (Os09g0440300) converts reactive aldehydes into corresponding carboxylic acids via oxidative reactions, thereby reducing the accumulation of reactive aldehydes and their toxic effects on cells under drought stress [35]. Aldehyde dehydrogenase (Os09g0440300) belongs to the family of NAD(P)-dependent enzymes, which catalyze the oxidation of endogenous and exogenous aldehydes to the corresponding carboxylic acids [15]. Studies in Arabidopsis showed that ALDH7B4 and ALDH3I1 play essential roles in aldehyde detoxification and reactive oxygen species (ROS) elimination, thus protecting plants from high-temperature damage [50]. The expression of alginase (Os10g0521000) is induced by ABA and is crucial in ABA-regulated stomatal closure, accelerating stomatal closure, and effectively reducing water loss [6, 50]. Malate synthase (Os04g0486950) is a key enzyme in the glyoxylate cycle, which is involved in the conversion of lipids to sugars and helps plants in nutrient redistribution [3]. Studies have demonstrated that malate synthase is a key gene involved in lipid mobilization and gluconeogenesis. Overexpression of the malate synthase gene from castor has been shown to enhance seed germination in Arabidopsis under high-temperature and salt stress conditions [1]. 1-Aminocyclopropane-1-carboxylic acid synthase (Os03g0727600) encodes an enzyme in the ethylene synthesis pathway. Under drought conditions, the expression of some genes in the ACS gene family (ACS1a/1b, ACS6a/6b, ACS9a) was up-regulated in quinoa at the root and aboveground, suggesting that the ACS gene family is involved in the drought stress response process [47]. Methylenetetrahydrofolate reductase (Os03g0815200) contributes to lignin accumulation [36, 40], which enhances plant tolerance to drought stress by reducing water infiltration and transpiration in the cell wall [20]. WRKY transcription factor (Os01g0826400) plays a crucial role in abiotic stress response. When the TaWRKY24 gene was silenced, wheat seedlings grew significantly slower than the normal control in drought conditions [45]. Overexpression of GhWRKY 41 increased drought resistance in transgenic tobacco when WRKY41 was isolated from land cotton and transferred to tobacco [4]. Acyl coenzyme A oxidase (Os11g0605500) enhances plant drought tolerance by promoting *β *-oxidation of fatty acids, reducing undesirable lipid peroxidation products (e.g., malondialdehyde), and maintaining membrane integrity [29]. Jasmonic acid biosynthesis, which requires acyl coenzyme A oxidase (Os11g0605500), has been closely associated with drought stress tolerance. Studies have shown that jasmonic acid enhances drought tolerance in peanuts [24], rice [7], soybean [25], and broccoli [42]. Peroxisomes are involved in lipid metabolism, photorespiration, reactive oxygen species detoxification and some phytohormone synthesis [28]. Peroxisome (S)-2-hydroxyacid oxidase GLO3 (Os04g0623500) is an important photorespiratory enzyme that plays a crucial role in regulating plant photosynthesis and enhancing plant acclimatization to stressful environments [19].

In addition to these gene-specific validations, we also reflected on the potential impact of our gene selection strategy. In this study, genes were filtered based on expression variance in order to reduce noise and computational complexity, as low-variance genes typically provide limited discriminatory power in predictive modeling [2]. This strategy has also been commonly applied in previous transcriptomic analyses [30]. To further minimize bias, we performed functional enrichment analysis, and the results showed that key drought-related biological processes, including osmotic stress response, water deficit adaptation, oxidoreductase activity, calcium binding, and cell wall modification, were still well represented after filtering. These findings indicate that our filtering step retained the major functional categories relevant to drought tolerance in rice. Nevertheless, we acknowledge that this approach may exclude some biologically important but low-variance genes. In future work, we plan to incorporate prior biological knowledge (e.g., known drought-responsive gene sets and pathway annotations) to complement variance-based filtering and ensure broader biological coverage.

## Conclusions

In this study, we integrated gene expression profile data and protein interaction network data to construct a comprehensive graph-based data and proposed an innovative DPGNNPAM algorithm. Concretely, the main advantages of our proposed DPGNNPAM are highlighted as several aspects: (1) an improved neighbor aggregation mechanism that effectively integrates homogeneous and heterogeneous information in graph-based data; (2) a class prototype-driven training approach that balances learning losses between majority and minority classes, enhancing predictive performance on imbalanced datasets; (3) robust performance in identifying drought-responsive genes in rice. Specifically, gene ontology enrichment and association analysis of the top 2,000 ranked genes revealed 17 candidate genes closely associated with drought stress, 12 of which were validated by evidence from existing literature.

Overall, the DPGNNPAM algorithm provides a novel perspective for uncovering latent patterns in biological data. In the future, we will further integrate multi-omics data, such as metabolomics and epigenomics, to enhance the algorithm’s capability in revealing complex molecular mechanisms and addressing broader biological challenges.

## Supplementary Information


Supplementary Material 1.
Supplementary Material 2.


## Figures and Tables

**Fig. 5:**
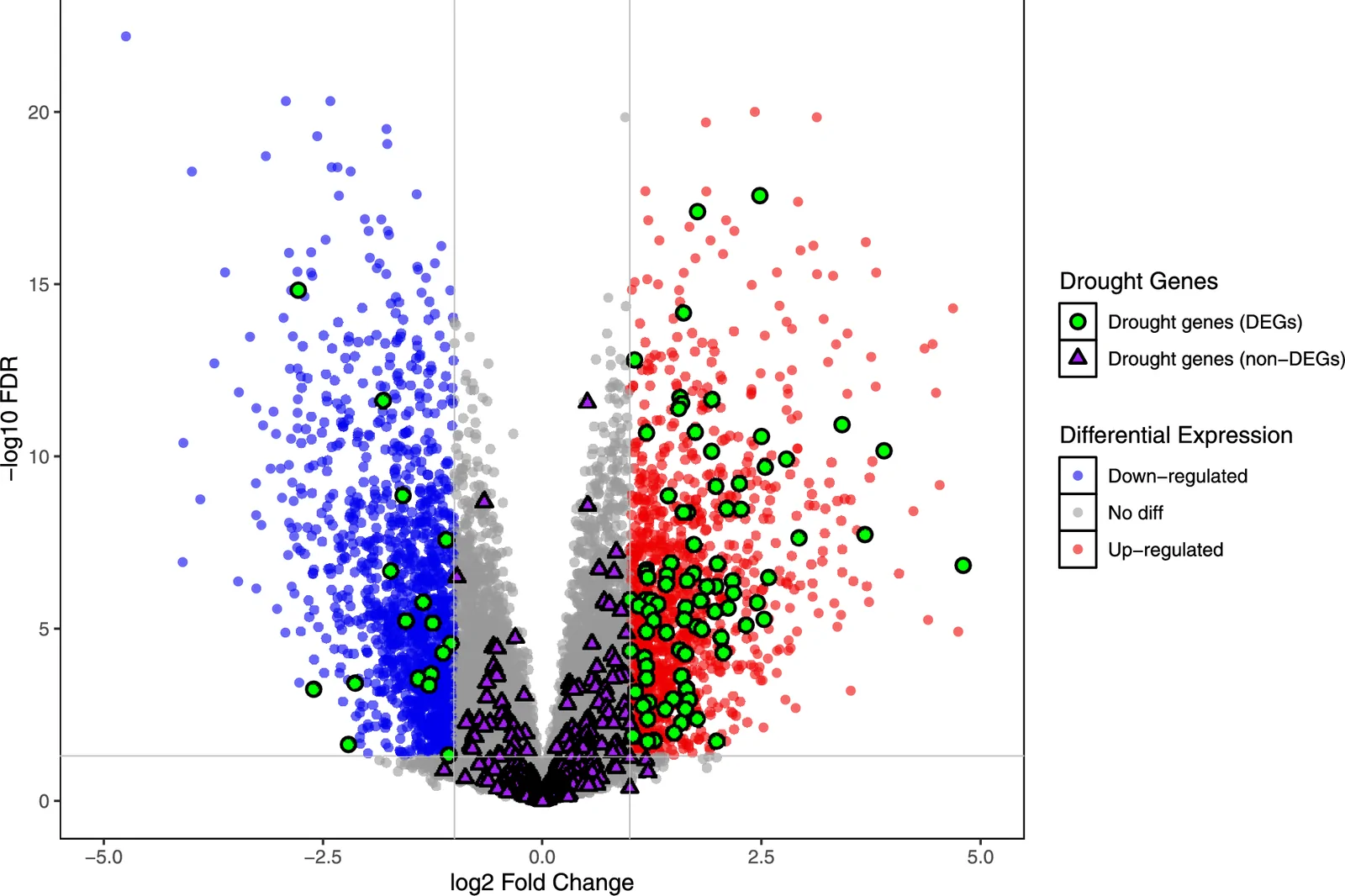
Volcano plot of differentially expressed genes (DEGs) under drought stress. Each dot represents a gene, with color indicating its expression pattern: blue for down-regulated, red for up-regulated, and gray for no significant change ($|log_2FC| \le 1$ and *FDR ≥ 0.05*). Known drought-responsive genes are highlighted using distinct symbols: green circles denote the 103 significantly differentially expressed drought genes ($|log_2FC| > 1$ and *FDR < 0.05*), while purple triangles represent the remaining 272 known drought-related genes that are not significantly differentially expressed (non-DEGs). The vertical lines indicate the fold-change threshold ($|log_2FC| = 1$), and the horizontal line marks the significance threshold (*FDR = 0.05*)

## Data Availability

All data generated or analysed during this study are included in this article.

## Appendix A: Gene ontology enrichment analysis

See Table 4.

**Table 4:** Enrichment analysis results of candidate drought-responsive genes

GO term	Count	Class	Enrichment	Description	*p* value
GO:0047484	7	BP	1.62	Regulation of response to osmotic stress	$3.53 \times 10^{-4}$
GO:0009605	63	BP	1.62	Response to external stimulus	$2.43 \times 10^{-4}$
GO:0006629	98	BP	2.06	Lipid metabolic process	$1.94 \times 10^{-11}$
GO:0009414	23	BP	6.15	Response to water deprivation	$1.35 \times 10^{-12}$
GO:0009266	39	BP	3.89	Response to temperature stimulus	$3.45 \times 10^{-13}$
GO:0009628	69	BP	2.67	Response to abiotic stimulus	$1.70 \times 10^{-13}$
GO:0009737	33	BP	4.78	Response to abscisic acid	$5.57 \times 10^{-14}$
GO:0009719	80	BP	2.76	Response to endogenous stimulus	$2.92 \times 10^{-16}$
GO:0006950	193	BP	2.04	Response to stress	$3.96 \times 10^{-21}$
GO:0043436	113	BP	3.02	Oxoacid metabolic process	$1.17 \times 10^{-25}$
GO:0012505	105	CC	1.35	Endomembrane system	$2.06 \times 10^{-3}$
GO:0005618	19	CC	3.5	Cell wall	$1.99 \times 10^{-6}$
GO:0005777	22	CC	3.89	Peroxisome	$4.61 \times 10^{-8}$
GO:0032991	50	CC	0.38	Protein-containing complex	$9.39 \times 10^{-18}$
GO:0005886	175	CC	2.25	Plasma membrane	$1.05 \times 10^{-23}$
GO:0016788	84	MF	1.77	Hydrolase activity, acting on ester bonds	$5.19 \times 10^{-7}$
GO:0022857	105	MF	1.74	Transmembrane transporter activity	$4.74 \times 10^{-8}$
GO:0005509	40	MF	2.65	Calcium ion binding	$2.50 \times 10^{-8}$
GO:0004601	29	MF	3.54	Peroxidase activity	$3.46 \times 10^{-9}$
GO:0016491	189	MF	2.43	Oxidoreductase activity	$1.15 \times 10^{-29}$

## Appendix B: Model robustness validation

### Label-shuffling experiment

To verify that our model learns biologically meaningful patterns rather than relying on spurious correlations or dataset biases, we conducted a label-shuffling experiment. In this controlled experiment, the gene labels (positive/negative for drought tolerance) were randomly shuffled across both training and test sets while preserving the original class distribution. All models were retrained and evaluated on this permuted dataset. The comparison results between the original and label-shuffled data are summarized in Table 5 below.

The results demonstrate a drastic performance drop across all informative metrics–particularly Precision, ROC-AUC, and PR-AUC–when labels are shuffled. We note that the relatively high accuracy under label shuffling is attributable to the strong class imbalance in the dataset; since the majority of samples are negative, a model that predominantly predicts the negative class can still achieve high accuracy even with randomized labels. This sharp performance decline confirms that the predictive power observed in the original setting stems from the model’s ability to capture genuine biological associations rather than overfitting or structural biases.

**Table 5:** Performance comparison on original and label-shuffled data

Model	Weighted F1	Accuracy	Precision	ROC-AUC	PR-AUC
shuffle_label	*0.7051 ± 0.0032*	*0.7657 ± 0.0227*	*0.1236 ± 0.0923*	*0.4814 ± 0.0244*	*0.1923 ± 0.0105*
DPGNNPAM	*0.8862 ± 0.0262*	*0.8987 ± 0.0186*	*0.9173 ± 0.0538*	*0.8759 ± 0.0292*	*0.7934 ± 0.0235*

### Performance under different data partitioning strategies

To further evaluate the model’s generalization ability and robustness to varying training set sizes, we assessed its performance under multiple data partitioning schemes, including stratified random sampling (2:1 and 4:1 train-test splits), k-fold cross-validation (3-fold and 5-fold), and a clustering-based strategy using the Leiden algorithm. The inclusion of the Leiden-based partitioning was motivated by the need to reduce potential information leakage that may arise from standard random splitting, particularly due to sequence or functional similarity among genes. By grouping genetically or functionally similar genes into the same cluster, this approach ensures that training and test sets are more independent, thereby providing a more realistic evaluation of model generalizability.

In the Leiden-based partitioning, we constructed a gene similarity network and applied the Leiden algorithm to identify densely connected modules. To ensure sufficient statistical power within each fold, we required that each module contain at least 1,000 genes. This process yielded six initial modules, with sample distributions across positive (drought-responsive), negative (non-responsive), and unlabeled genes summarized in Table 6.

**Table 6:** Distribution of gene samples across Leiden-based clustering

Module	Positive	Negative	Unlabeled
0	153	542	5670
1	80	297	5260
2	83	238	3108
3	38	205	2387
4	10	80	1420
5	11	115	1056

Since the last two modules (Modules 4 and 5) contained relatively few positive and negative samples, we manually merged them. The resulting five modules used for five-fold cross-validation are shown in Table 7.

**Table 7:** Distribution of gene samples across Leiden clusters after merging modules 4 and 5

Module	Positive	Negative	Unlabeled
0	153	542	5670
1	80	297	5260
2	83	238	3108
3	38	205	2387
4	21	195	2476

Using these five merged modules, we conducted five-fold cross-validation under the Leiden partitioning scheme. The results, summarized in Table 8, show that the model maintains competitive performance under Leiden-based partitioning, despite the more stringent and biologically informed data separation. While there is a moderate decrease in certain metrics–particularly in ROC-AUC, likely due to reduced inter-cluster similarity–the model still achieves high weighted F1 and PR-AUC values, indicating robust precision and recall for the positive class. These findings confirm that our model generalizes well even when preventing potential data leakage through structurally aware partitioning.

**Table 8:** Comparison of model performance under different data partitioning strategies

	Weighted F1	Accuracy	Precision	ROC-AUC	PR-AUC
2:1 (random)	0.8766 ± 0.0126	0.8889 ± 0.0107	0.8934 ± 0.0454	0.8489 ± 0.0153	0.7610 ± 0.0314
3-fold	0.8650 ± 0.0154	0.8834 ± 0.0152	0.9711 ± 0.0273	0.8386 ± 0.0100	0.7675 ± 0.0110
4:1 (random)	0.8862 ± 0.0262	0.8987 ± 0.0186	0.9173 ± 0.0538	0.8759 ± 0.0292	0.7934 ± 0.0235
5-fold	0.8845 ± 0.0135	0.8963 ± 0.0107	0.9249 ± 0.0337	0.8564 ± 0.0301	0.7647 ± 0.0568
Leiden (ReVar)	0.8529 ± 0.0252	0.8430 ± 0.0349	0.8341 ± 0.0809	0.6476 ± 0.0684	0.7066 ± 0.0671
Leiden (DPGNNPAM)	0.8712 ± 0.0292	0.8897 ± 0.0271	0.9529 ± 0.0920	0.7867 ± 0.0860	0.6911 ± 0.1642

Overall, the consistent performance across diverse partitioning strategies, including the conservative Leiden-based approach, demonstrates the robustness and generalizability of our model, especially in scenarios where data independence is strictly enforced.

## Appendix C: Differential expression analysis of candidate genes

To comprehensively evaluate gene response patterns under drought stress, we performed genome-wide differential expression analysis. As shown in Fig. 5, the volcano plot displays the expression change patterns of all detected genes under drought treatment. Among the known 375 drought-resistance genes (remaining in the network after data preprocessing), 103 showed significant differential expression, with 86 upregulated and 17 downregulated. This result validates the responsiveness of these known drought-responsive genes in our experimental system.

We next focused on the 17 candidate drought-responsive genes predicted by our computational method. Their differential expression profiles under drought stress are presented in Table 9.

**Table 9:** Differential expression analysis of the 17 candidate drought-responsive genes

Gene ID	logFC	AveExpr	t	*p* value	Adj. *p* val	Expression
Os04g0486950	4.3618	4.3605	10.0879	4.5893e−16	7.3831e−14	Up
Os04g0623500	1.8175	4.1788	7.3170	1.4904e−10	4.4441e−09	Up
Os05g0163700	1.5997	5.7499	7.2179	2.3312e−10	6.5643e−09	Up
Os11g0605500	1.2787	5.2562	6.7239	2.1258e−09	4.5955e−08	Up
Os09g0440300	1.7632	7.7465	6.3330	1.1877e−08	2.0558e−07	Up
Os10g0521000	1.5898	4.1987	3.7014	0.00038545	0.0017251	Up
Os04g0106300	0.9946	5.3173	4.6421	1.2825e−05	9.2372e−05	No diff
Os10g0390500	0.8261	5.5910	3.1655	0.00216813	0.0074656	No diff
Os03g0195100	0.8608	1.4673	2.6163	0.01056665	0.0279671	No diff
Os04g0578000	0.8172	1.9500	2.5716	0.01191397	0.0308139	No diff
Os06g0347100	1.8986	4.7374	2.3340	0.02202371	0.0512103	No diff
Os06g0345200	0.3132	3.9209	1.4242	0.15814432	0.2508384	No diff
Os01g0826400	0.4660	3.3514	1.0542	0.29485795	0.4108767	No diff
Os06g0196700	0.3031	3.7131	0.9592	0.34026752	0.4576699	No diff
Os03g0727600	0.1200	0.8891	0.5974	0.55188914	0.6584659	No diff
Os01g0954000	0.1021	3.6109	0.2071	0.83642951	0.8841001	No diff
Os03g0815200	0.0594	7.3567	0.1835	0.85482259	0.8974167	No diff

The differential expression analysis reveals that 6 out of the 17 candidate genes (35.3%) show significant up-regulation under drought stress conditions (*FDR ≤ 0.05* and *|logFC| ≥ 1*). Among the up-regulated genes, the log fold changes range from 1.28 to 4.36. The statistical support for these findings is strong, with false discovery rates (FDRs) for the up-regulated candidates all below 0.05, and half of them achieving exceptionally low FDRs (below $10^{-8}$). This independent transcriptomic evidence demonstrates that a substantial proportion of our computationally predicted genes are indeed transcriptionally responsive to drought conditions, thereby supporting the biological relevance of our predictions.

## Code availability

The source code and data set can be downloaded freely from https://github.com/lucky0172/DPGNNPAM.
